# Tumor suppression by small molecule inhibitors of translation initiation

**DOI:** 10.18632/oncotarget.598

**Published:** 2012-08-25

**Authors:** Limo Chen, Bertal H Aktas, Yibo Wang, Xiaoying He, Rupam Sahoo, Nancy Zhang, Severine Denoyelle, Eihab Kabha, Hongwei Yang, Revital Yefidoff Freedman, Jeffrey G Supko, Michael Chorev, Gerhard Wagner, Jose A Halperin

**Affiliations:** ^1^ Harvard Medical School; ^2^ Brigham and Women's Hospital; ^3^ Massachusetts General Hospital

**Keywords:** Translation, eIF4F, eIF4E, ternary complex, eIF2

## Abstract

Translation initiation factors are over-expressed and/or activated in many human cancers and may contribute to their genesis and/or progression. Removal of physiologic restraints on translation initiation causes malignant transformation. Conversely, restoration of physiological restrains on translation initiation reverts malignant phenotypes. Here, we extensively characterize the anti-cancer activity of two small molecule inhibitors of translation initiation: #1181, which targets the eIF2-GTP-Met-tRNA_i_ ternary complex, and 4EGI-1, which targets the eIF4F complex. *In vitro*, both molecules inhibit translation initiation, abrogate preferentially translation of mRNAs coding for oncogenic proteins, and inhibit proliferation of human cancer cells. *In vivo*, both #1181 and 4EGI-1 strongly inhibit growth of human breast and melanoma cancer xenografts without any apparent macroscopic- or microscopic-toxicity. Mechanistically, #1181 phosphorylates eIF2α while 4EGI-1 disrupts eIF4G/eIF4E interaction in the tumors excised from mice treated with these agents. These data indicate that inhibition of translation initiation is a new paradigm in cancer therapy.

## INTRODUCTION

Eukaryotic translation is regulated by the eukaryotic translation initiation factors (eIFs), features of mRNAs, and signaling pathways. Two multi-protein complexes are rate limiting for translation initiation. The eIF4F complex is formed by the scaffolding protein eIF4G the RNA helicase eIF4A, and the mRNA cap binding protein eIF4E. The ternary complex (TC) is formed by the interaction of eIF2 with GTP and initiator methionine tRNA (Met-tRNA_i_). The eIF4F complex binds to the mRNA 5’ cap structure and associates with 40S ribosomal subunit, the TC, and other translation initiation factors to form the 48S pre-initiation complex that scans the mRNA 5’ untranslated region (5'UTR) to locate the AUG initiation codon.

Translation initiation plays a critical role in cell growth and malignant transformation [1-6]. In quiescent cells eIF4E-binding proteins (4E-BPs) restrict the abundance of the eIF4F complex, while phosphorylation of eIF2α on S51 restricts the availability of the TC. In proliferating cells, phosphorylation of 4E-BPs reduces their affinity for eIF4E and increases the abundance of the eIF4F complex [7]. Similarly dephosphorylation of eIF2α allows for eIF2B catalyzed GDP-GTP exchange on the eIF2-GDP, and increases the abundance of the TC. Malignant transformation is associated with a preferential increase in the translation of mRNAs encoding for growth factors and/or oncogenic proteins. These mRNAs contain long and highly structured 5'UTRs, multiple upstream untranslated open reading frames (uORFs), or other features that reduce their translational efficiency and render them highly dependent on the activity of translation initiation factors [4, 5]. Housekeeping proteins, on the other hand, are coded for by efficiently translated mRNAs with short and simple 5'UTRs. Unrestricted translation, therefore, preferentially increases the expression of oncogenic proteins and promotes malignant transformation [8-11]. Consistently, restricting translation initiation by reducing the abundance of either eIF4F or TC preferentially decreases the expression of oncogenic proteins thereby reverting malignant phenotypes [12-14].

Levels of eIF4E [8, 15-19], eIF4G [6, 20] and eIF4A [21, 22] are frequently up-regulated in human cancers. Notably, in head and neck and breast cancers, levels of eIF4E correlate with disease progression and poor prognosis [8, 10, 18, 23]. Similarly, in non-Hodgkin's lymphomas and thyroid carcinomas, levels of eIF2α correlate with disease status [24-26].

In experimental models of cancer forced expression of eIF4E [27], of a constitutively active but non-phosphorylatable mutant of eIF2α (eIF2α-S51A) [11] or of initiator Met-tRNA_i_ [28] transforms immortalized fibroblasts. Conversely, decreasing the levels of eIF4E by treatment with eIF4E anti-sense RNA or its activity by ectopic expression of 4E-BPs partially reverses the transformed phenotypes [9, 12, 13]. Pharmacologically, treatment with eIF4E anti-sense RNA or agents that induce phosphorylation of eIF2α inhibits translation initiation and proliferation of cancer cells *in vitro*, and reduces tumor growth in animal models of human cancers [29-31]. Similarly, inhibitors of the mammalian target of rapamycin (mTOR), which reduces phosphorylation of 4E-BPs [32, 33], exert anti-cancer activity *in vitro* [14, 34] and *in vivo* [35]. Finally, small molecules such as pateamine A and silvestrol - reduce the activity of the eIF4F complex by modulating the activity of eIF4A thereby suppressing translation initiation [36-38]. Taken together, these data indicate that translation initiation is a promising new paradigm and an attractive target for the development of anti-cancer agents.

We previously reported the identification of the translation initiation inhibitor 4EGI-1, which binds to eIF4E and thereby disrupts eIF4E/eIF4G interaction [39]. Additionally we reported on the development of #1181 [40], which causes eIF2α phosphorylation [40] thereby inhibiting cap-dependent translation and proliferation of cancer cells. These findings suggested that 4EGI-1 and #1181 are suitable probes for testing the hypothesis that small molecule inhibitors of translation initiation are mechanism specific anti-cancer agents.

Here we report the anti-cancer efficacy, mode of action, pharmacokinetics, and toxicity profiles of 4EGI-1 and #1181. Both agents inhibit translation initiation and preferentially abrogate expression of oncogenic proteins *in vitro*. *In vivo*, 4EGI-1 and #1181 strongly inhibit tumor growth in xenograft models of human breast and melanoma cancers with no sign of macroscopic- or microscopic-toxicity at therapeutic doses. In xenograft tumors, #1181 phosphorylates eIF2α and 4EGI-1 disrupts eIF4G/eIF4E interaction. Both agents inhibit expression of oncogenic proteins such as cyclin E, cyclin D1, c-Myc and Bcl-2 *in vivo*. We conclude that translation initiation can be pharmacologically targeted for cancer therapy.

## RESULTS

### In vitro characterization of #1181 and 4EGI-1

Based on the high prevalence of breast cancer and melanoma, we screened approximately 20 different melanoma and breast cancer cell lines using a combination of IC_50_ *in vitro* (Supplemental Table S1) and tumorigenicity in nude mice as final selection criteria. Consequently, human melanoma (CRL-2813) and breast cancer (MCF-7 and CRL-1500) cells were chosen for testing the *in vitro* and *in vivo* efficacy of #1181 and 4EGI-1.

**Figure 1:**
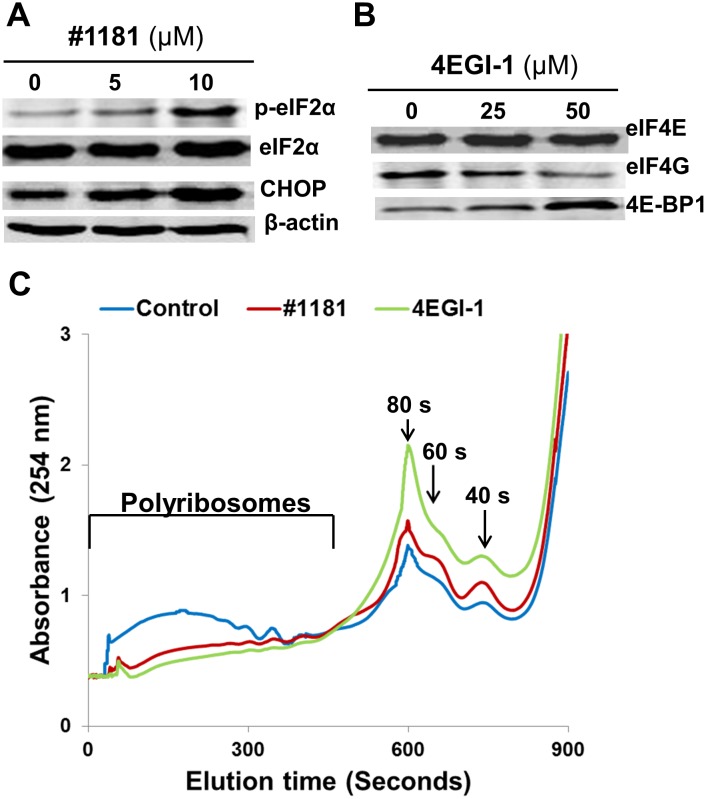
#1181 and 4EGI-1 inhibit translation initiation A) CRL-2813 human melanoma cells were treated with the indicated concentrations of #1181, cell lysates were probed with antibodies specific to S51 phosphorylated eIF2α, total eIF2α, CHOP and β-Actin. B) CRL-2813 cells were treated with the indicated concentrations of 4EGI-1, eIF4E was pulled-down from the lysates using M^7^GDP Sepharose cap affinity column. Proteins were eluted from the column with free M^7^GDP and probed with antibodies specific to eIF4G, eIF4E or 4E-BP1. C) Cells were treated with 10 μM #1181 or 50 μM 4EGI-1 for 3 hours, cytoplasmic extracts were overlaid on 15-60% sucrose gradient and subjected to ultracentrifugation. The gradients were eluted from the bottom under constant monitoring at 254 nm.

**Figure 2:**
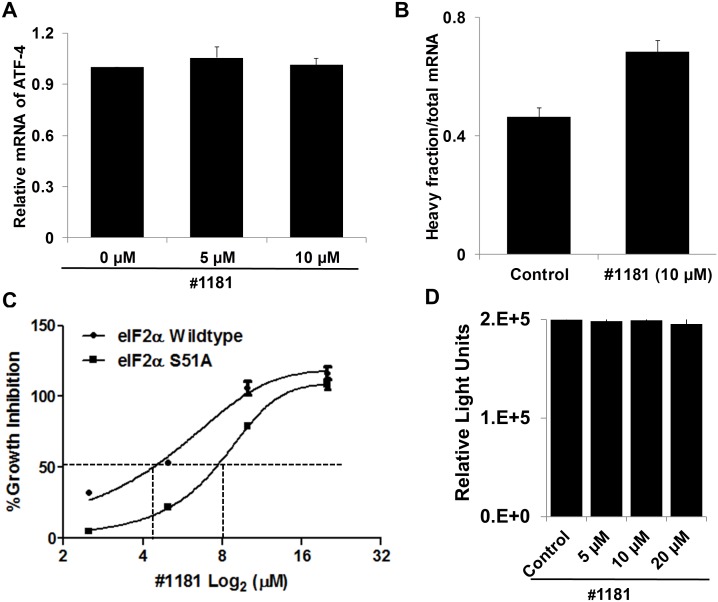
#1181 increases the recruitment of ATF-4, a downstream effector eIF2α phosphorylation, to heavy polysomes but does not inhibit protein synthesis in cell-free extracts A) Total RNA was prepared from CRL-2813 cells incubated for 3 hours in the presence or absence of #1181. ATF-4 mRNA levels were determined by QRT-PCR. B) The distribution of ATF-4 mRNA along the polysome profile was determined using fractioned RNA from polysome profiles shown in Figure 1C. C) The wild type eIF2α or S51A mutant eIF2α expressing PC3 cells were treated with #1181 in indicated concentrations [48]. The growth inhibition was measured by SRB assay. D) The *in vitro* translation assay was performed according to the protocol of Retic Lysate IVTTM Kit (Ambion, cat. #AM1200). The effect of #1181 on the translation efficiency of luciferase RNA (Promega, cat. #L4561) was determined by measuring the luminescence with Wallac Envision Reader.

Inhibition of translation initiation in vitro: In mechanistic assays, #1181 induced phosphorylation of eIF2α (Figure 1A). As shown previously, 4EGI-1 reduced the association of eIF4G with eIF4E (Figure 1B) [39]. Both compounds shifted the polysome profile of cancer cells from heavy to light polysomes or free ribosomal subunits (Figure 1C), clearly demonstrating that #1181 and 4EGI-1 inhibit translation initiation. Furthermore, #1181 induced expression of C/EBP homology protein (CHOP) and activating transcription factor 4 (ATF-4)- two downstream effectors of eIF2α phosphorylation (Figures 1A, 2A, and 2B). In mechanistic assays, #1181 inhibited cancer cell proliferation in an eIF2α phosphorylation dependent manner. This is evidenced by the fact that replacing endogenous eIF2α with recombinant eIF2α S51A mutant rendered the cancer cells resistant to inhibition of cell proliferation by #1181 as compared to cells in which endogenous eIF2α was replaced with recombinant wild type eIF2α (Figure 2C). Consistent with demonstration that in intact cells, #1181 induces phosphorylation of eIF2α via Ca^++^ release from internal stores [40], this compound had no direct inhibitory effect on protein synthesis in cell-free lysates (Figure 2D).

**Figure 3:**
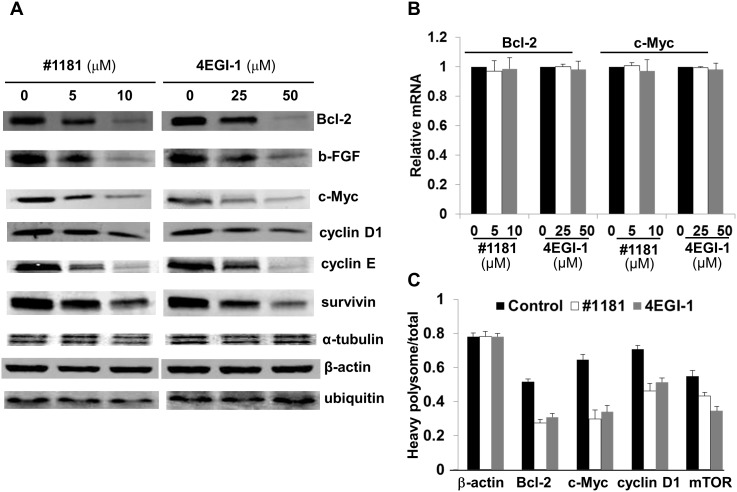
#1181 and 4EGI-1 preferentially inhibit expression of oncogenic proteins A) CRL-2813 human melanoma cells were treated with the indicated concentrations of #1181 or 4EGI-1, lysates were prepared and probed with antibodies specific to Bcl-2, b-FGF, c-Myc, Cyclin D1, Cyclin E, Survivin, α-Tubulin β-Actin, and Ubiquitin. B) RNA was prepared from similarly treated cells and levels of cyclin D1, cyclin E, survivin, b-FGF, c- Myc, Bcl-2 and β-actin mRNAs were determined. Shown are mRNA levels for c-myc and bcl-2 relative to controls. Data for other mRNAs are shown in the Supplementary Figure S4. C) The level of various oncogenic and housekeeping mRNAs in the polysome fractions of cells treated with vehicle, #1181 and 4EGI-1 were determined by QRT-PCR.

Expression of most proteins involved in cell proliferation and malignant transformation is translationally controlled and is highly dependent on the activity of translation initiation factors. To determine if #1181 and 4EGI-1 translationally downregulate expression of oncogenic proteins, we performed Western blot (WB) and quantitative real time PCR (QRT-PCR) analyses of lysates from CRL-2813 human melanoma cells treated with #1181, 4EGI-1 or vehicle (DMSO). Figure 3A shows that both compounds significantly reduced the expression of c-Myc, Cyclin D1, Cyclin E, Bcl-2, bFGF and Survivin while the expression of housekeeping proteins such as β-Actin, α-Tubulin and Ubiquitin was not affected (for quantitation of WB data see Supplemental Figure S1). Down-regulation of most oncogenic proteins was likely translational because the compounds had minimal effects on the levels of the respective mRNAs (Figure 3B and Supplemental Figure S2). In a few instances, and only at high concentrations of #1181 or 4EGI-1, accumulation of oncogenic mRNAs was reduced (Supplemental Figure S2). These findings are consistent with the view that inhibitors of translation initiation preferentially affect the expression of oncogenic proteins. This was further confirmed by QRT-PCR measuring the distribution of mRNAs in the polysome fractions by RT-PCR (Figure 3C). The shift in polysome profile caused by treatment with #1181 or 4EGI-1 (Figure 1C) was associated with a preferential shift in the distribution of oncogenic mRNAs from heavy to light polysomes or polysome-free fractions.

**Figure 4:**
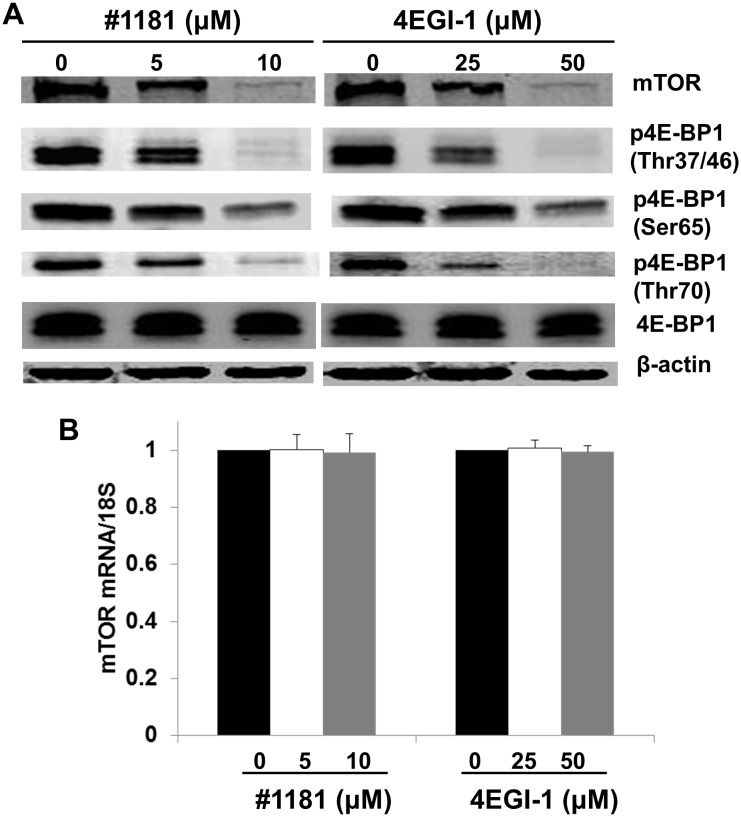
Translation initiation inhibitors abrogate mTOR expression and 4E-BP1 phosphorylation A) CRL-2813 cells were treated with the indicated concentrations of #1181 or 4EGI-1. Cell lysates were probed with antibodies specific to mTOR, 4E-BP1 phosphorylated (p4E-BP1) on the indicated residues, total 4E-BP1, and β-actin. B) Cells were treated as in A and level of mTOR mRNA in treated cells relative to control cells was determined by QRT-PCR.

Both #1181 and 4EGI-1 translationally reduced the expression of mTOR protein (Figure 4A and Supplemental S3) and the phosphorylation of 4E-BP1 (Figures 4A) with no effect on the levels of mTOR mRNA (Figure 4B). This is consistent with the known pleiotropic effects of the mTOR [41]. It must be noted; however, that 4EGI-1 inhibits eIF4E/eIF4G interaction independently of 4E-BP1 binding to eIF4E [39]. Furthermore, neither #1181 nor 4EGI-1 impinge directly on the PI3-K Akt pathway that is activated in the MCF-7 and CRL-1500 breast cancer cells, or the B-raf/Erk pathway that is activated in CRL-2813 melanoma cell line, as demonstrated by the experiment depicted in Supplemental Figures S4A and S4B.

**Figure 5:**
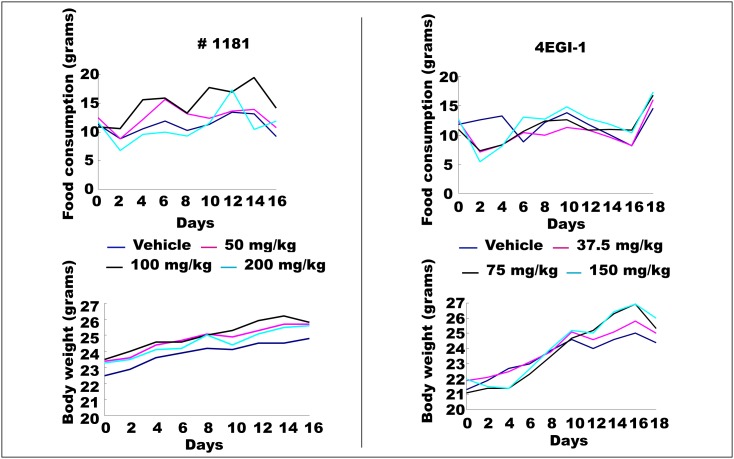
Maximum tolerated dose (MTD) assay for #1181 and 4EGI-1 MTD was assessed by injecting groups of 5 male and 5 female nude mice with different intra-peritoneal (i.p.) doses of each compound for 5 consecutive days followed by observation for additional 15 days, in accord with NIH protocols. At the concentrations used, injection of either compound did not result in significant weight loss, reduced daily food intake, behavioral changes or any other observable sign of toxicity.

**Figure 6:**
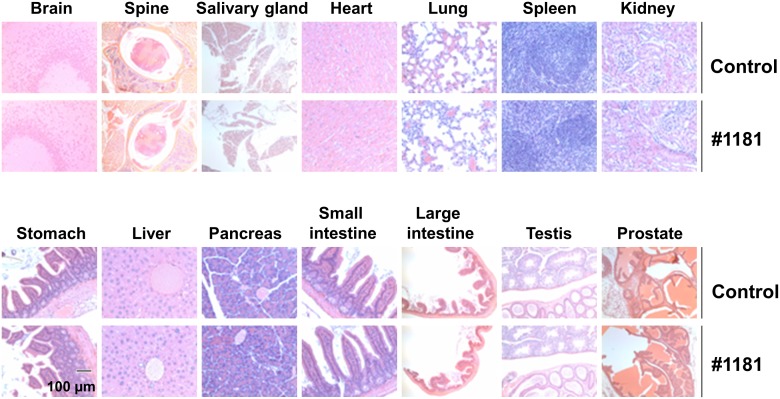
#1181 displays no organ toxicity Nude mice (5 mice each group) were treated i.p. with 175 mg/kg b.i.d. #1181 or vehicle b.i.d. for 21 days. At the end of treatment, mice were euthanized, major organs were harvested, stored in Bouin's solution, and sectioned for microscopic examination. The displayed slides are representative of the sample pool available.

In vivo characterization of #1181 and 4EGI-1: To assess the *in vivo* anti-cancer activity of #1181 and 4EGI-1 we attempted to determine the maximum tolerated dose (MTD) of both agents by injecting groups of 5 male and 5 female nude mice with different intraperitoneal (i.p.) doses of each compound for 5 consecutive days. This was followed by observation for an additional 14 days. At the concentrations used, neither compound caused significant weight loss, behavioral changes, reduced daily food intake, or any other overt toxicity (Figure 5). Limited solubility of compounds in aqueous buffers precluded further dose escalation to determine MTD. The long-term organ toxicity was evaluated by treating mice i.p. for 21 days with daily injections of the highest compound dose used in the efficacy studies described below. Necropsy pathological analysis of these mice did not reveal any macro- or microscopic evidence of organ toxicity (Figure 6 and Supplemental S5).

Plasma concentration-time profiles of the two compounds in mice treated i.p. with a 25 mg/kg of #1181 in 200 μL of corn oil or 50 mg/kg of 4EGI-1 in 25 μl of DMSO are presented in Supplemental Figure S6A. Both compounds were rapidly absorbed from the peritoneal cavity, with peak concentrations C(max) = 5.2 μM and 139 μM occurring at t(max) = 52 min and 43 min for #1181 and 4EGI-1, respectively. Plasma concentrations of both compounds decayed in a mono-exponential manner with half-life t_1/2_ = 1.6 h for #1181 and 3.4 h for 4EGI-1. Values of the apparent total body clearance were CL/F = 90.4 and 2.4 ml/min/kg for #1181 and 4EGI-1, respectively. Furthermore, as shown in Supplemental Figures S6B and S6C, the plasma concentration of #1181 or 4EGI-1 exhibited excellent dose dependence. The doses and treatment regimens were chosen based on these findings. Due to its lower plasma exposure and shorter half-life, #1181 was administered twice daily, (b.i.d.) while 4EGI-1 was administered once a day (q.d.).

**Figure 7:**
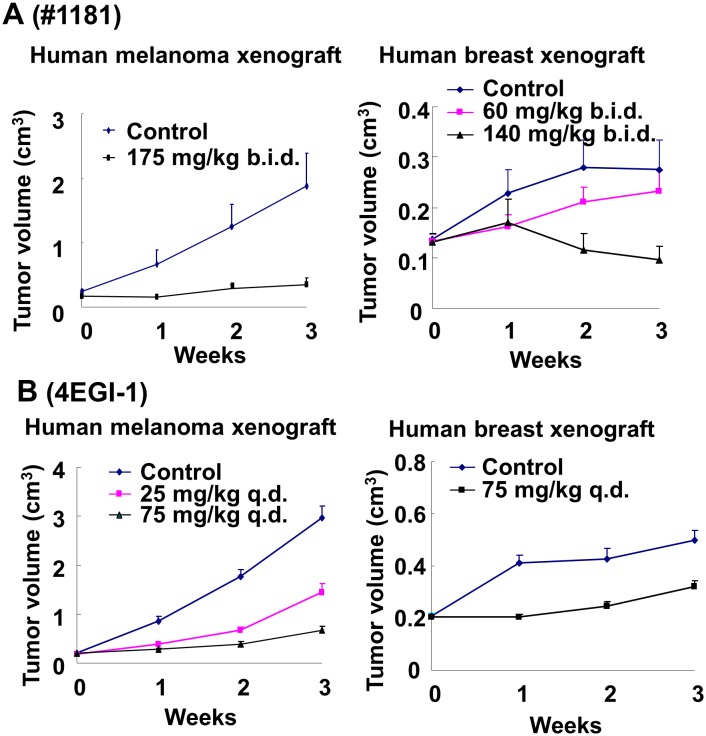
*In vivo* efficacy of translation initiation inhibitors for cancer therapy Mouse carrying xenografted human melanoma or breast cancer (~150 mm^3^) were randomly distributed to control and treatment groups and treated i.p. with the indicated daily doses of #1181 b.i.d. (A) or 4EGI-1 q.d. (B). Tumor dimensions were measured weekly and tumor volumes were calculated.

Inhibition of tumor growth: Mice bearing CRL-2813 human melanoma tumors (apparent volume of ≈ 200 mm^3^) were randomized into control and treatment groups. Compound #1181 was dissolved in corn oil and injected i.p. 175 mg/kg b.i.d in 125 μl corn oil 12 h apart. Control animals received, by the same regimen, the same daily amount of corn oil. Figure 7A (left panel) and Supplemental Figure S7A (left panel) show that #1181 totally abolished human melanoma xenograft tumor growth.

To determine if #1181 inhibits growth of mammary tumors, estrogen-dependent MCF-7 human breast cancer cells were inoculated into the fat pad of the 4^th^ inguinal mammary gland of female mice implanted with slow release 17-β-estradiol pellets in the subscapular region. Animals bearing ≈150 mm^3^ tumors were randomized into control and treatment groups and administered #1181 i.p. at 60 or 140 mg/kg b.i.d. in 100 μl of corn oil 12 h apart or the same regimen of daily amount of corn oil. Figure 7A (right panel) and Supplemental Figure S7A (right panel) show that #1181 caused a dose-dependent inhibition of MCF-7 human breast cancer tumor growth; the highest dose induced a ≈ 30% regression of the tumors.

To determine the anti-tumor efficacy of 4EGI-1, mice bearing ≈ 200 mm^3^ human melanoma tumors were randomized into three groups and injected i.p. with 25 or 75 mg/kg q.d. 4EGI-1 in 12.5 μl DMSO or 12.5 μl DMSO. Figure 7B (left panel) and Supplemental Figure S7B (left panel) show that 4EGI-1 significantly, and dose-dependently, inhibited human melanoma xenograft tumor growth. Similarly, mice bearing orthotropic ≈200 mm^3^ CRL-1500 derived xenograft breast tumors were treated i.p. with 75mg/kg q.d. of 4EGI-1 in 12.5 μl DMSO, with control animals receiving the same daily amount of DMSO. Figure 7B (right panel) and S7B (right panel) show that 4EGI-1 caused a significant inhibition of CRL-1500 human breast tumor growth.

### Molecular analysis of tumors

**Figure 8:**
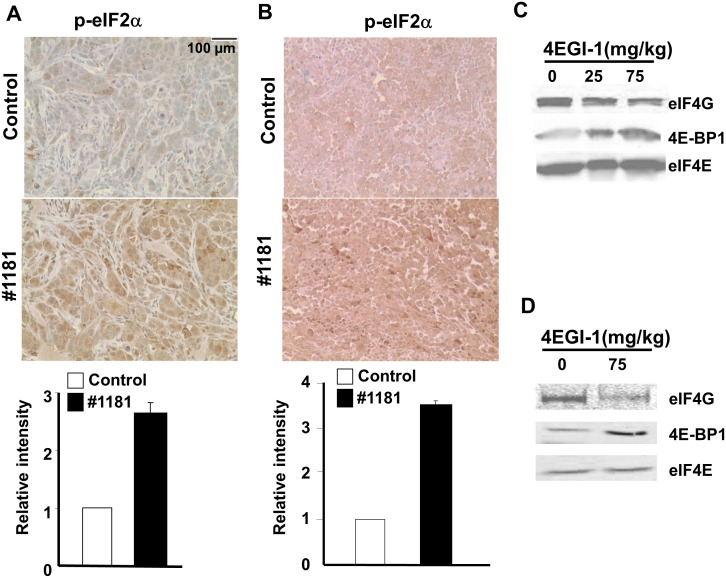
#1181 causes phosphorylation of eIF2α while 4EGI-1 disrupts eIF4E/eIF4G interactions *in vivo* A) and B) human CRL-2813 melanoma (A) and MCF-7 breast (B) xenograft carrying mice were treated i.p. with #1181 (175 mg/kg and 120 mg/kg, b.i.d., respectively) for three days, tumors were excised, formaldehyde fixed, and stained with antibodies specific to S51 phosphorylated and total eIF2α. Bar graphs show ratio of phosphorylated to total eIF2α. The displayed section are representative of numerous sequential slices obtained. C and D) human CRL-2813 melanoma (C) and CRL-1500 breast (D) xenograft carrying mice were treated i.p. with indicated doses of 4EGI-1 for three days, tumors were excised, lysed, and eIF4E was pulled-down using M^7^GDP affinity column. Eluted proteins were blotted with antibodies specific to eIF4G, eIF4E or 4E-BP1.

To assess their *in vivo* mode of action, we evaluated the effects of #1181 and 4EGI-1 on the phosphorylation of eIF2α and disruption of eIF4E/eIF4G interaction in the excised tumors. Paraffin embedded sections of melanoma and breast tumors excised from mice treated with #1181 or vehicle were stained with antibodies specific to S^51^ phosphorylated or total eIF2α. Figures 8A and 8B show that #1181 significantly increased the phosphorylation of eIF2α in the tumors. The *in vivo* effects of compound 4EGI-1 on the formation of eIF4F complex were investigated by pulling-down eIF4E from tumor lysates by 7-methylguanosine diphosphate (M^7^GDP)-Sepharose affinity chromatography followed by WB analysis of eIF4E, eIF4G and 4E-BP1. Tumors from mice treated with 4EGI-1 showed a reduced association of eIF4E with eIF4G and an increased association of eIF4E with 4E-BP1 (Figures 8C and 8D).

**Figure 9:**
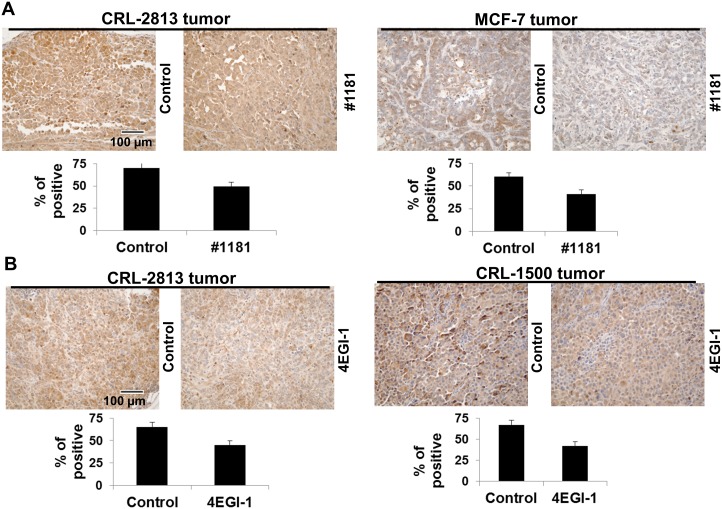
#1181 and 4EGI-1 down-regulate the phosphorylation of 4E-BP1 in tumors The sections from the excised tumors taken from the efficacy studies were stained with phospho-4E-BP1 (Thr37/46) antibody (Cell Signaling, cat. #2855). The data was quantified by ProImage software. The displayed section are representative of numerous sequential slices obtained.

**Figure 10:**
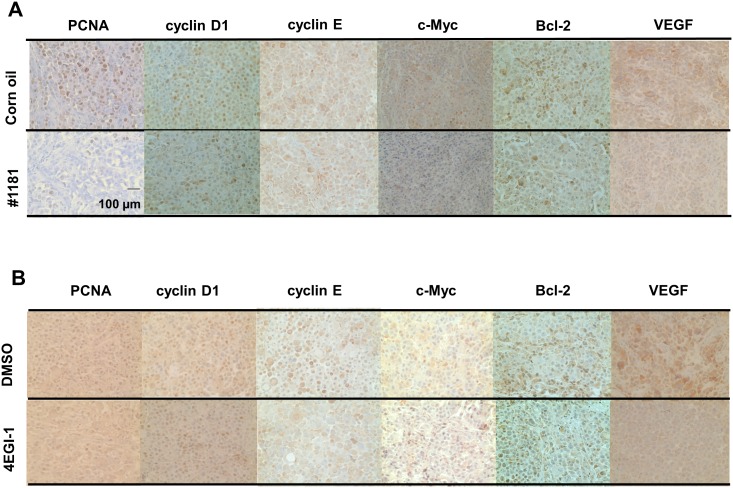
#1181 and 4EGI-1 downregulate expression of oncogenic proteins in xenograft models of human melanoma #1181-treated (A) or 4EGI-1-treated (B) CRL-2813 human melanoma xenografts were immunostained with antibodies specific for PCNA, cyclin D1, cyclin E, c-Myc, bcl-2, and VEGF. For the immunohistochemistry images, pictures were taken in three random fields from each sample section. Figure S13 depicts quantification of the data. The displayed section are representative of numerous sequential slices obtained.

We also stained tumor sections with antibodies specific to phosphorylated 4E-BP1 and oncogenic and growth promoting proteins such as cyclin D1, cyclin E, c-Myc, Bcl-2, and VEGF. Consistent with their *in vitro* activities both #1181 and 4EGI-1 reduced phosphorylation of 4E-BP1 (Figure 9) and the expression of oncogenic proteins in the tumors (Figure 10 and Supplemental Figures S8-S10). Both agents significantly reduced the expression of proliferating cell nuclear antigen (PCNA, Supplemental Figures S8-S10) without any effect on the expression of B-raf, phosphorylated Erk1/2 or Akt, or increase in the proportion of apoptotic cells as determined by TUNEL assay (Supplemental Figure S11).

## DISCUSSION

Excessive activation and/or overexpression of translation initiation factors cause malignant transformation and maintenance of transformed phenotypes *in vitro* and *in vivo* [8-11, 27]. Translation initiation factors are also implicated in the genesis, maintenance and progression of human cancers [8, 16-19] suggesting that translation initiation may be an attractive target for cancer therapy. However, the lack of potent and specific small molecule inhibitors of translation initiation has hampered the experimental assessment of whether the translation initiation machinery can be pharmacologically targeted for therapeutic purposes.

The work reported here provides direct evidence that inhibition of translation initiation with either #1181 or 4EGI-1 abrogates tumor growth in two animal models of human cancer. Compound #1181 induces eIF2α phosphorylation, which reduces the abundance of the TC. Compound 4EGI-1 inhibits eIF4E/eIF4G protein-protein interaction, which reduces the abundance of the eIF4F complex [39, 40]. Both compounds abrogate tumor growth and cause comparable down-regulation of oncogenic proteins *in vivo*. Importantly, histo-pathological and hematological analysis of treated tumor-bearing mice demonstrated that neither 4EGI-1 nor #1181 cause any sign of overt toxicity. Our demonstration that both compounds recapitulate *in vivo* their biological activities *in vitro* validates the concept that the anti-cancer effect of both compounds is most likely mediated by inhibition of translation initiation.

One of the oncogenic proteins translationally down regulated by both 4EGI-1 and #1181 is mTOR, which couples PI3K/Akt signaling with assembly of the eIF4F complex by inducing phosphorylation of 4E-BP1. This in-turn reduces 4E-BP1's affinity to eIF4E and makes it available for eIF4F assembly. This suggests the 4EGI-1 and #1181 may create a feedback loop that further potentiates their inhibitory effect, and potentially highlights another major advantage of small molecule inhibitors of translation initiation for cancer therapy.

Our findings indicate that by depriving cancer cells of the oncogenic proteins critical for maintaining their transformed phenotype, small molecules #1181 and 4EGI-1 target cancer cells at their “Achilles heel” [42, 43]. Targeting the expression of multiple oncogenic proteins that the cancer cells are addicted to represents a new paradigm in cancer therapy and has distinct advantages over both conventional genotoxic therapies and recently developed therapeutic agents that target a single oncogene/survival factor [44]. The latter therapeutic approach is compromised because cancer cells develop resistance to the drug by either activating redundant/alternate pathway(s) to compensate for the loss of the targeted molecule or sustain mutations that renders the primary target refractory to the therapeutic agent [45]. Significant progress in resolving the structure of translation initiation factors [46, 47] as well as discovery of novel inhibitory agents [48], should significantly aid discovery and development of translation initiation inhibitors. In conclusion, inhibition of translation initiation is a promising complement to the prevailing anti-cancer therapies because it is aimed at the convergent point of oncogenic and proliferative pathways. This paradigm represents a solid rational for developing and testing small molecule inhibitors of translation initiation in clinical studies for anti-cancer therapy.

## MATERIALS AND METHODS

### Cell growth assay

All cell lines were purchased from American Type Culture Collection (ATCC), maintained per ATCC protocols and utilized within 6 months of thawing each vial. The inhibition of cell growth was measured by the sulforhodamine B (SRB) assay [31].

### Polysome profiles

Polysome profiles were obtained by the sucrose density gradient centrifugation method [29].

### Animal studies

CRL-2813 (451Lu, B-raf V600E mutant) melanoma cells were injected subcutaneously (2.5 × 10^5^ cells in 0.1 ml of 50% matrigel) into 6-week-old male nude mice (Charles River Laboratories). Tumor-bearing mice were randomized into control and treatment groups, treated intra-peritoneal (IP) with the vehicle, 4EGI-1 (75 and 25 mg/kg q.d. in 12.5 μl DMSO) or #1181 (175 mg/kg b.i.d. in 125 μl corn oil), Tumor volumes were calculated as in [30] and results analyzed by Student's t-test.

Female mice implanted with 90 day slow release 17-β-eastradiol pellet in the subscapular region were inoculated with MCF-7 (HTB-22, PI3Kα mutant) or CRL-1500 (ZR75-1, PTEN deficient) human breast cancer cells into the 4^th^ inguinal gland. Tumors were allowed to grow 150 mm^3^ size, animals were randomly distributed to control and treatment groups. Mice bearing MCF-7 tumors were treated IP with 140 or 60 mg/kg b.i.d. #1181 in 100 μl corn oil or corn oil alone. Mice bearing CRL-1500 xenografts were treated i.p. with 75 mg/kg q.d. 4EGI-1 in 1.5 μl DMSO or DMSO alone. All animal studies were approved by the Harvard Medical School Institutional Animal Care and Use Committee and conducted in accordance with the Animal Care and Use Committee approved protocols.

### Immunohistochemistry

Formalin-fixed, paraffin-embedded tumor sections were immunostained with antibodies and counter-stained with hematoxylin. List and sources of antibodies are given in Supplemental Table S1. Pictures were taken with Nikon (ECLIPSE) microscope via Nikon (Plan Fluor) lenses by Nikon (TE2000-E) camera. Images were acquired in JPEG format using SPOT Advanced software and staining was quantified with ProImage software. All antibodies used for these studies are listed in Supplemental Table S2.

### Western blot analysis

The WB analysis was performed as described [49]. For eIF2α phosphorylation, cells were treated with either #1181 or DMSO for 1 hour. For analysis of other proteins, cells were incubated for 8 hours in the presence or absence of each compound.

### Quantitative Real-time PCR analysis

Cells were incubated for 8 hours in the presence or absence of the compounds. The FastLane Cell SYBR Green Kit (Qiagen) was used to purify and analyze the mRNA levels with an Applied Biosystems Thermocycler. RT-PCR quantitation of the mRNAs relative to β-actin mRNA was done using the ΔΔCT method. Sequence-validated QuantiTec probes for bcl-2, bFGF, survivin, mTOR, cyclin D1, cyclin E, c-Myc, and β-actin purchased from Qiagen Bio-technology were used for these mRNAs. QRT-PCRs were also performed to determine the polysome profile shifts for β-actin, mTOR, Bcl-2, c-Myc, cyclin D1 and ATF-4 using total RNA isolated from polysome fractions.

### m7GTP Pull-Down assay

CRL-2813 cells were treated with 4EGI-1 or DMSO for 8 hours, harvested by centrifugation and lysed. The interaction of eIF4E with eIF4G was determined by the m^7^GTP Pull-Down assay as described [39]. For *in vivo* pull-down experiments, extracts of tumors excised from mice treated with either 4EGI-1 or DMSO were similarly analyzed.

## Supplementary Figures and Tables




