# Supplementary material for: Tumor suppression by small molecule inhibitors of translation initiation
Source: Oncotarget. 2012 Aug 25;3(8):869–81. doi: 10.18632/oncotarget.598 (PMC3478463; doi:10.18632/oncotarget.598)
Supplement: Supplementary file 1 [file oncotarget-08-869-s001.pdf]

TUMOR SUPPRESSION BY SMALL MOLECULE INHIBITORS OF TRANSLATION INITIATION - Limo Chen et al

SUPPLEMENTARY MATERIALS AND METHODS:

Maximum tolerated dose (MTD). 6-week-old nude mice (Charles River Laboratories) were divided into four groups (5 males and 5 females in each group). #1181 was dissolved in 125 µl corn oil administered at the concentrations of 50 mg/kg, 100 mg/kg and 200 mg/kg twice daily. 4EGI-1 was dissolved in 12.5 µl DMSO and administered at the concentrations of 37.5 mg/kg, 75 mg/kg and 150 mg/kg. Each compound was administered by IP injection for 5 consecutive days. At the end of the treatment the mice were closely monitored for additional 15 days, according with NIH protocols. Food consumption and body weight were measured every two days. Mouse behavior and hair loss were included in the observations.

Pharmacokinetic studies. Pharmacokinetics of #1181 and 4EGI-1 was assessed by analyzing blood samples employing high performance liquid chromatography (HPLC) coupled electrospray ionization mass-spectrometry (ESI-MS). The lower limits of quantitation of these assays were 0.070 µM for #1181 and 0.056 µM for 4EGI-1.

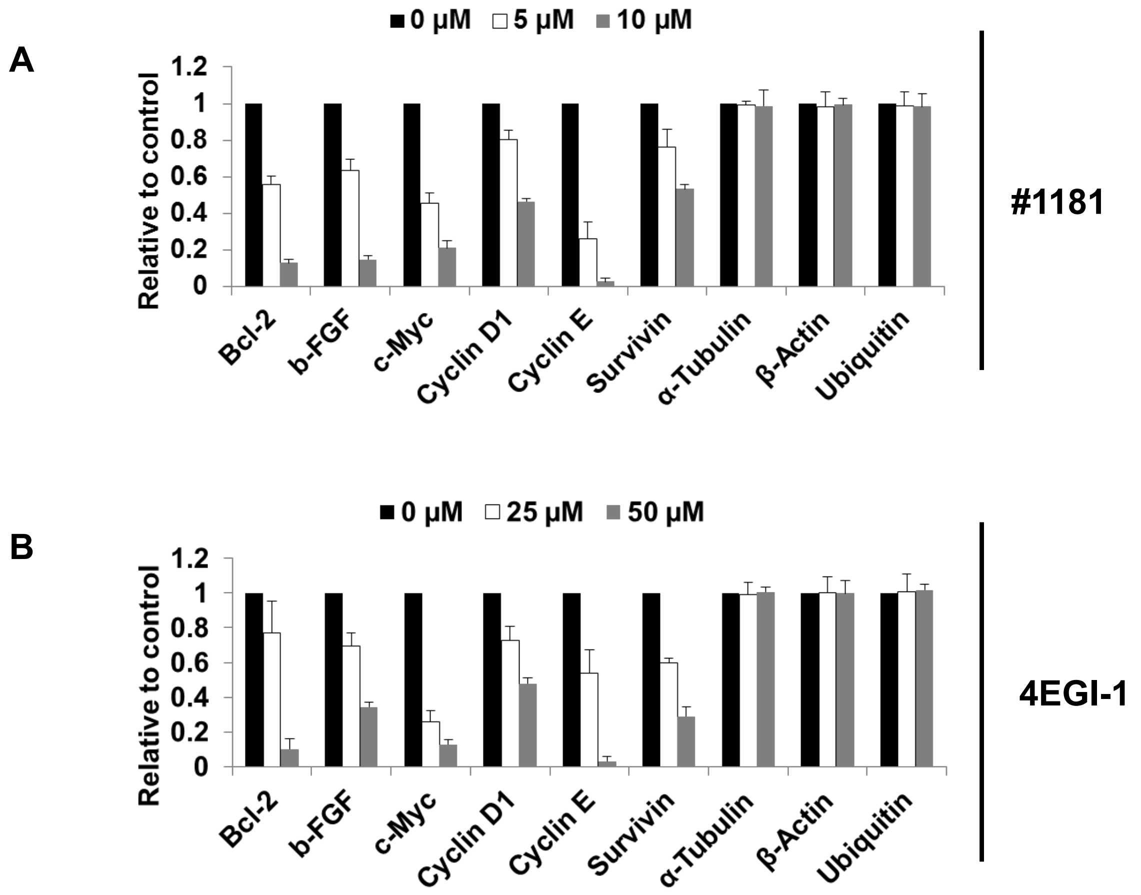

Figure S1: The quantification of Western blotting data for Figure 3A. A) Treatment with #1181. **B**) Treatment with 4EGI-1. Blots were quantified using Odyssey software. The signal of three blots from three independent experiments was measured. Shown are the means, error bars denote SEM.

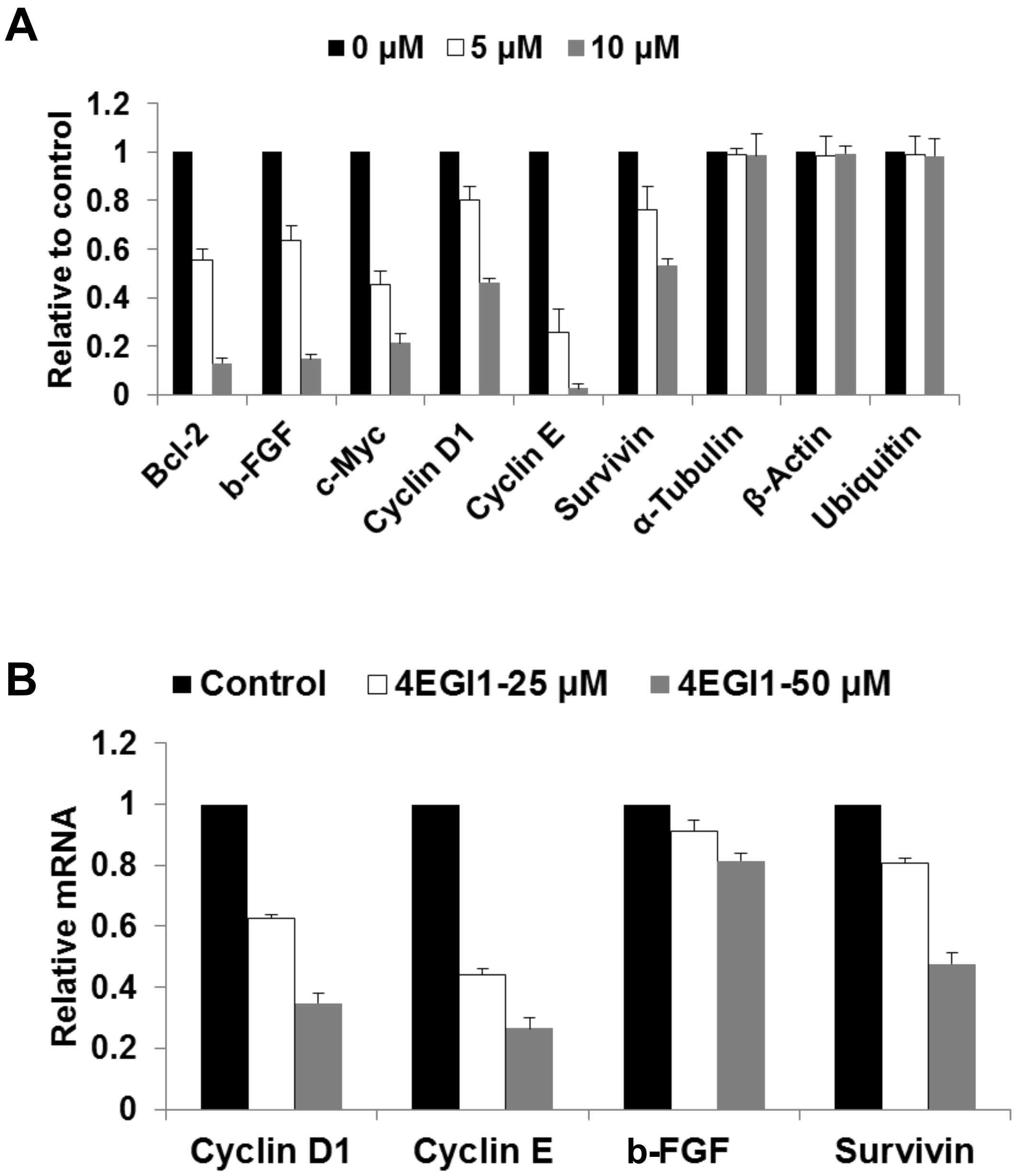

Figure S2: Effect #1181 (A) and 4EGI-1 (B) on the mRNA levels of additional oncogenes (A and B). CRL-2813 cells were incubated for 8 hours in the presence or absence of the compounds. The FastLane Cell SYBR Green Kit (Qiagen) and an Applied Biosystems thermocycler were used to purify and analyze the mRNA levels with sequence-validated QuantiTec probes from Qiagen. Real-time PCR quantitation of the mRNA relative to β-actin was done by using the ΔΔCT method.

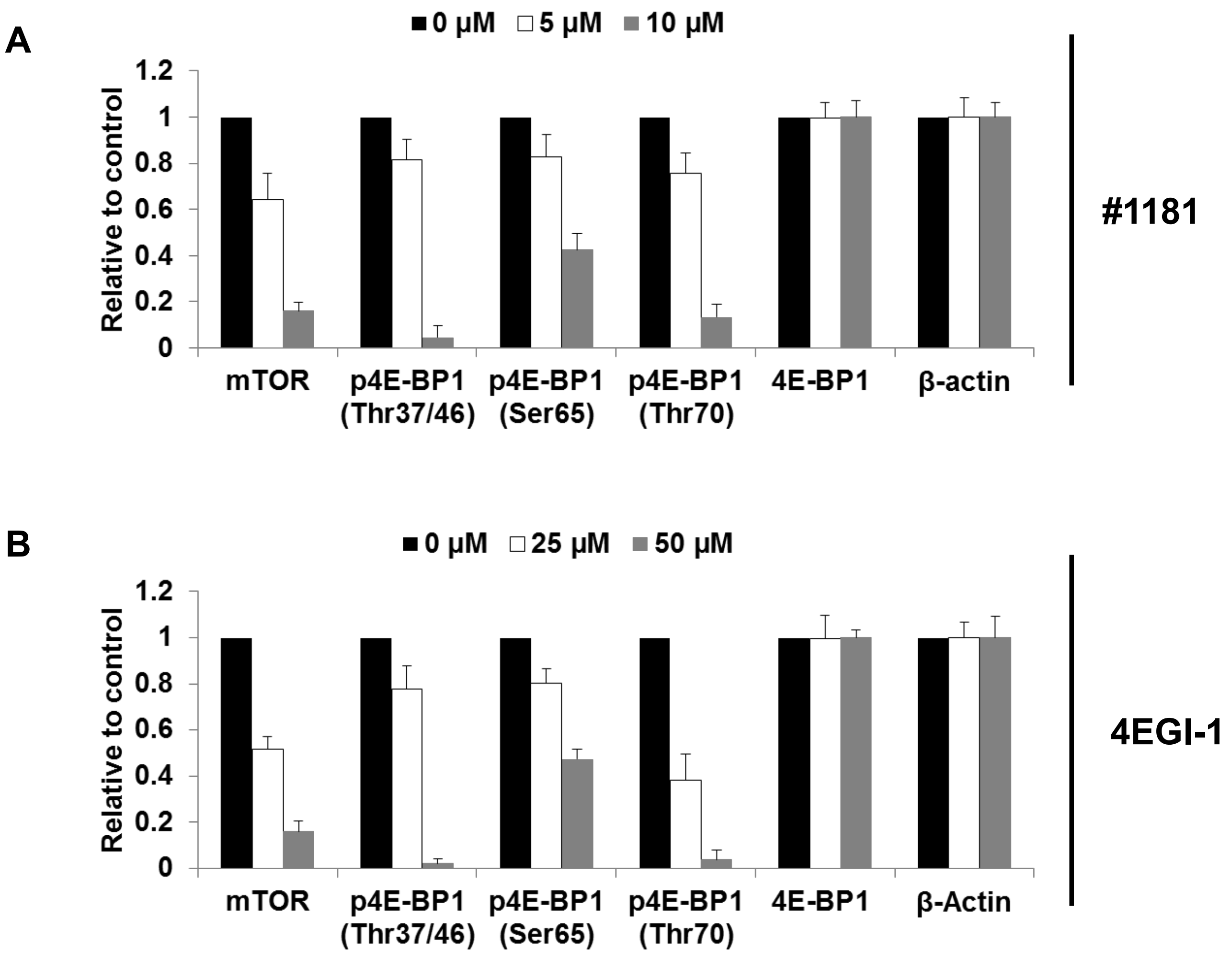

Figure S3: The quantification of Western blotting data for Figure 4A. Blots were quantified using Odyssey software. The bars represent average signals of three blots from three independent experiments, error bars denote SEM.

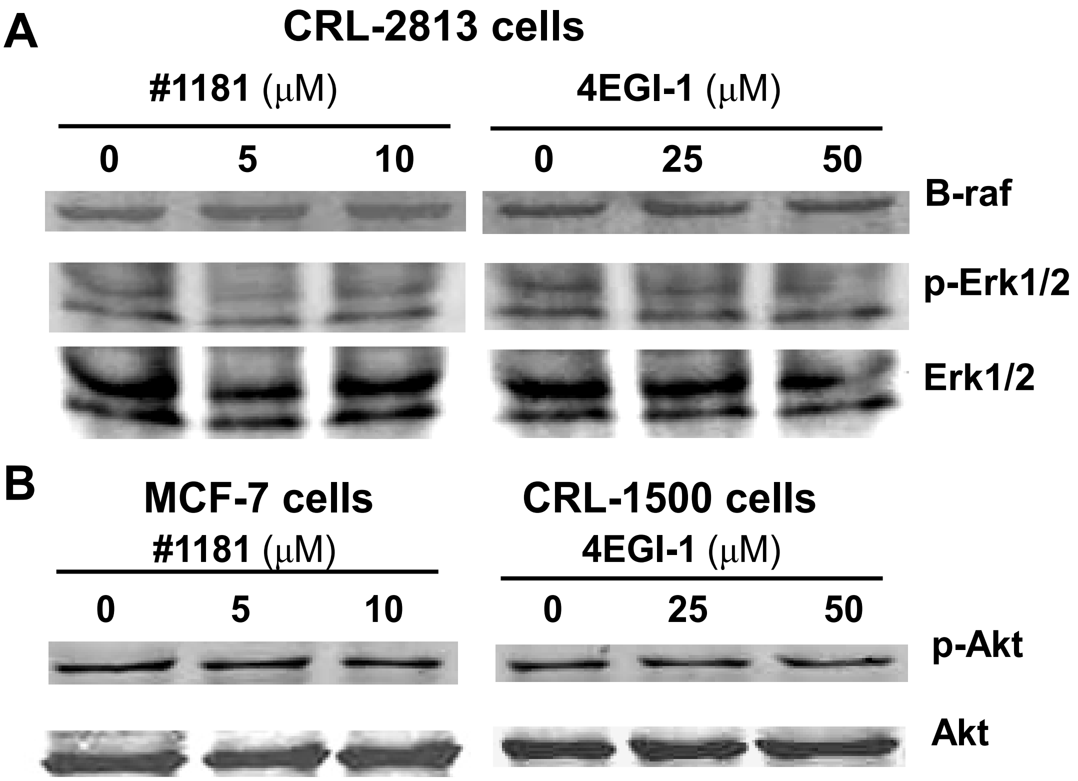

Figure S4: *In vitro* evidence of the action of #1181 and 4EGI-1 independent of B-raf, Erk1/2 and Akt. A) Human CRL-2813 melanoma cancer cells were exposed to #1181 and 4EGI-1 at the indicated concentrations for 8 hours. Cell lysates were analyzed by WB using specific antibodies. **B)** MCF-7 and CRL-1500 breast cancer cells were treated with #1181 and 4EGI-1 and cell lysates were probed with antibodies specific to Akt and phospho-Akt.

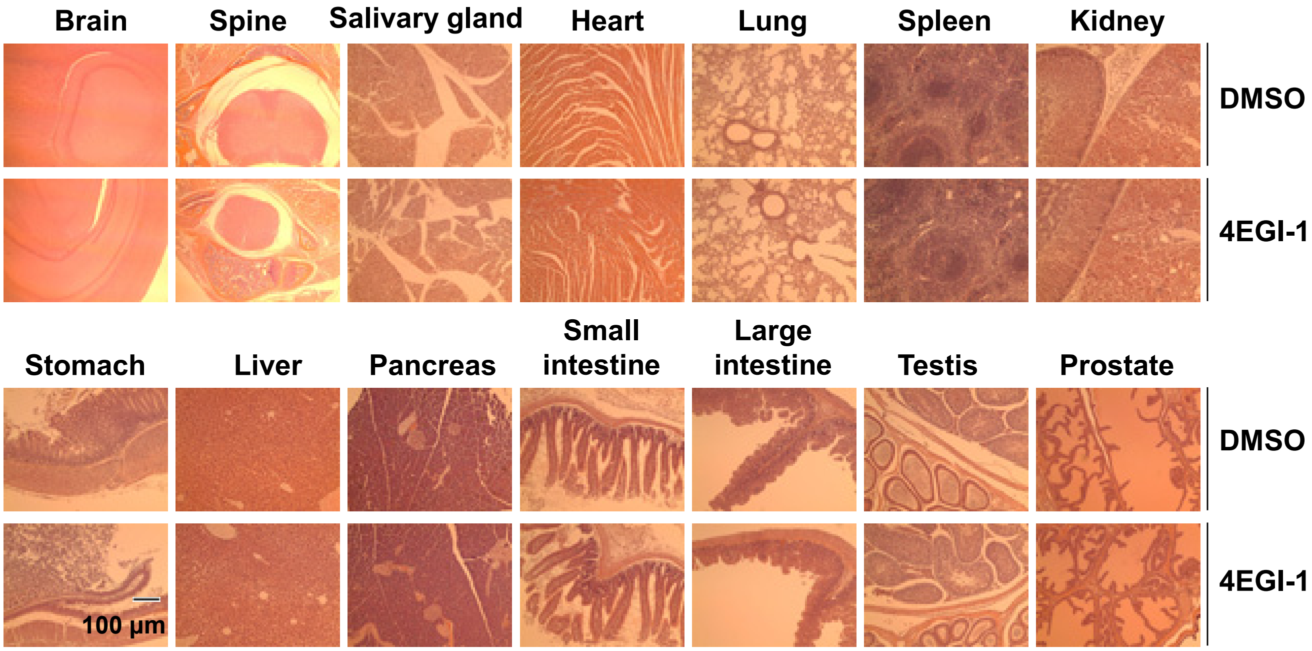

Figure S5: 4EGI-1 displays no organ toxicity. Nude mice (5 mice each group) were treated daily with 75mg/kg 4EGI-1 or vehicle for 21 days and then euthanized. Major organs were harvested, stored in Bouin's solution and sectioned for microscopic examination. The representative fields are shown here.

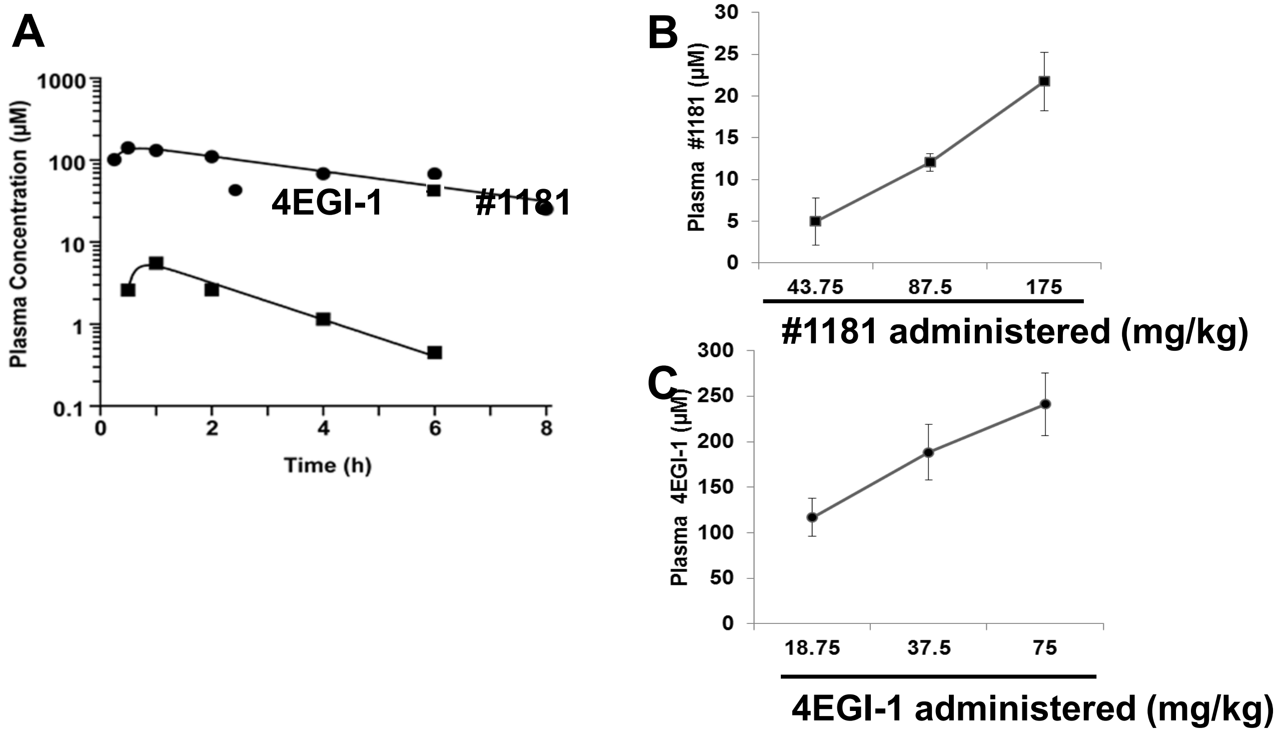

Figure S6: The pharmacokinetics of #1181 and 4EGI-1. Analytical methods based upon high performance liquid chromatography coupled with electrospray ionization mass spectrometry were developed and validated for the determination of compounds #1181 and 4EGI-1 in mouse plasma. **A**) Plasma concentration-time profiles of the two compounds were determined by treating mice (5 mice each group) via IP injection with 25 mg/kg of compound #1181 in 200 μL of corn oil or 50 mg/kg of compound 4EGI-1 in 25 μl of DMSO. **B and C**) Plasma concentration-dose profiles of the two compounds were determined by treating mice (5 mice each group) via IP injection with the indicated concentrations. The plasma concentration 1 h after dosing with #1181 or 4EGI-1 are shown.

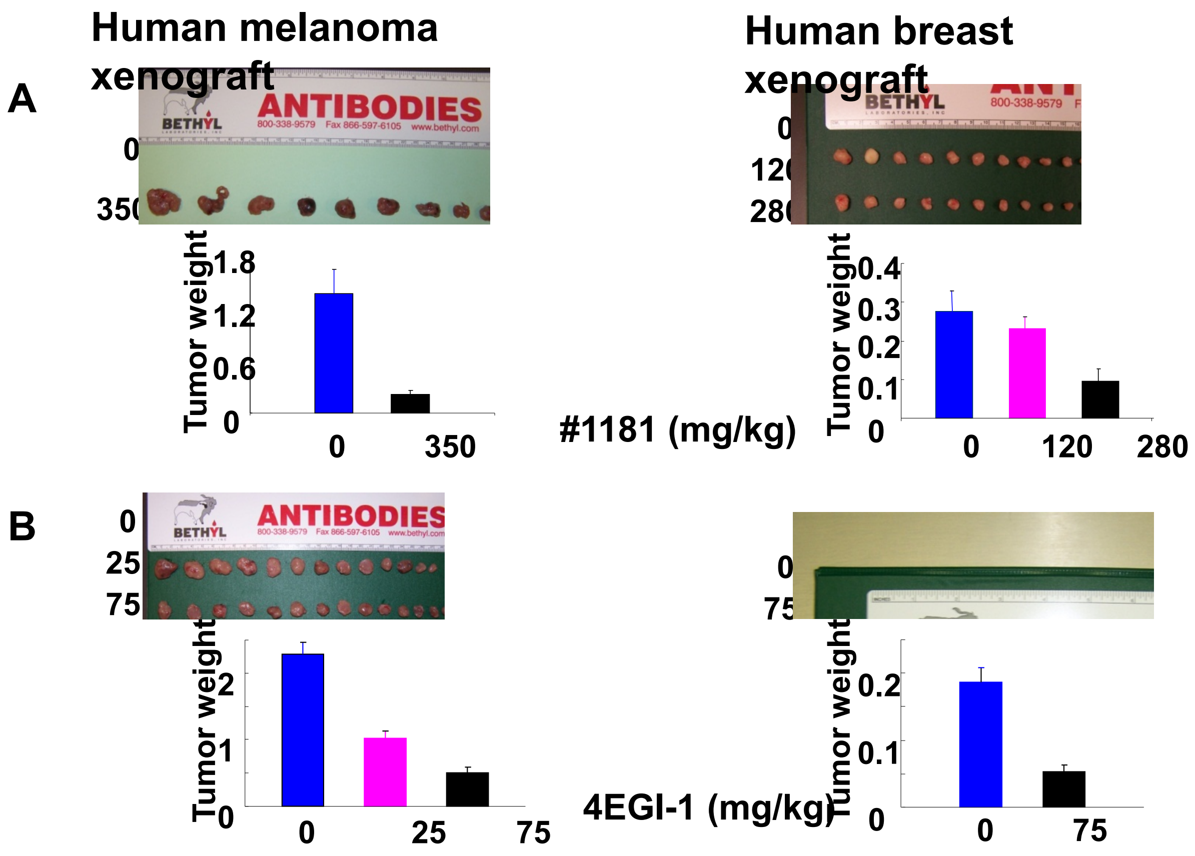

Figure S7: The photographs and the final weight of tumors. At the end of the efficacy experiment mice were sacrificed by CO_2 asphyxiation, tumors were removed, photographed (upper panels), and weighed (bottom panels).

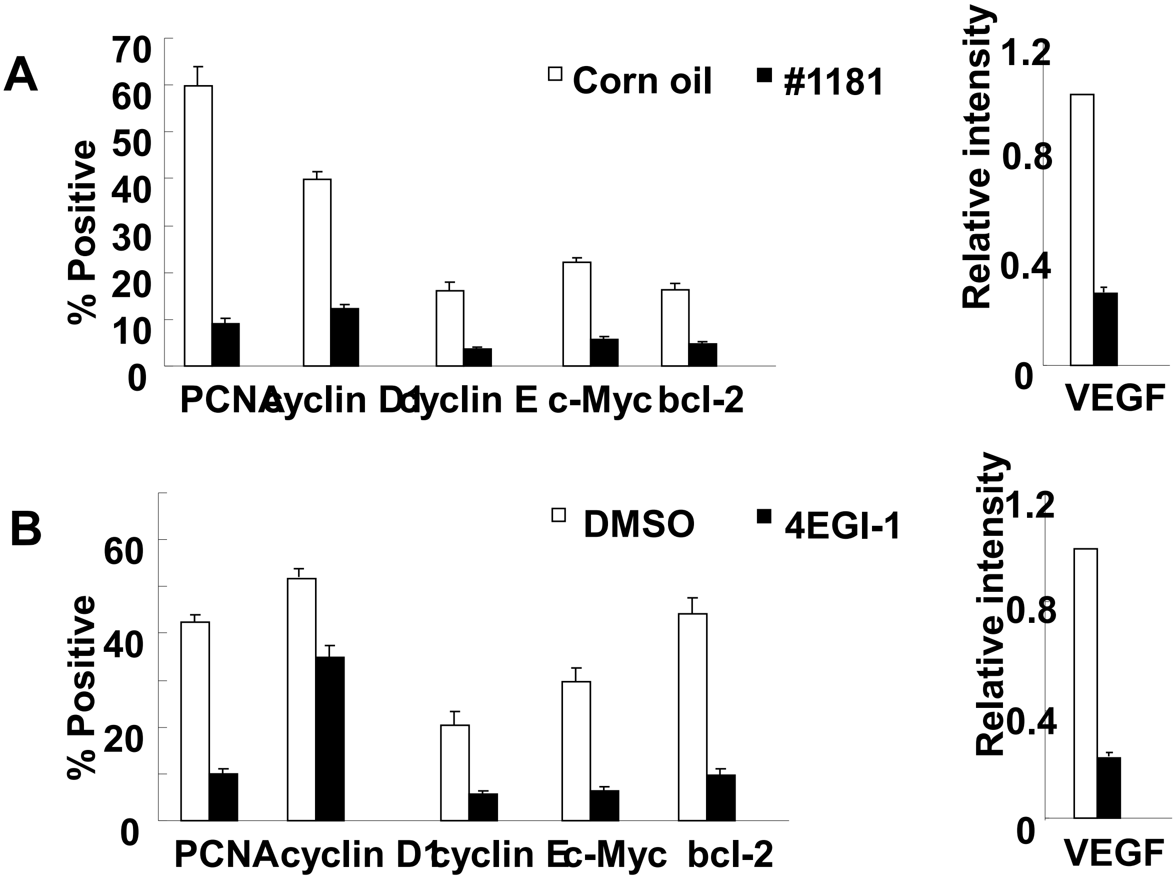

Figure S8: The quantification of immunohistochemistry for Figure S12. For quantifying the immunohistochemistry images, pictures were taken in three random fields from each sample section. The scoring was determined using ProImage software. The bars represent average signals of three independent fields and at least five different mice, error bars denote SEM.

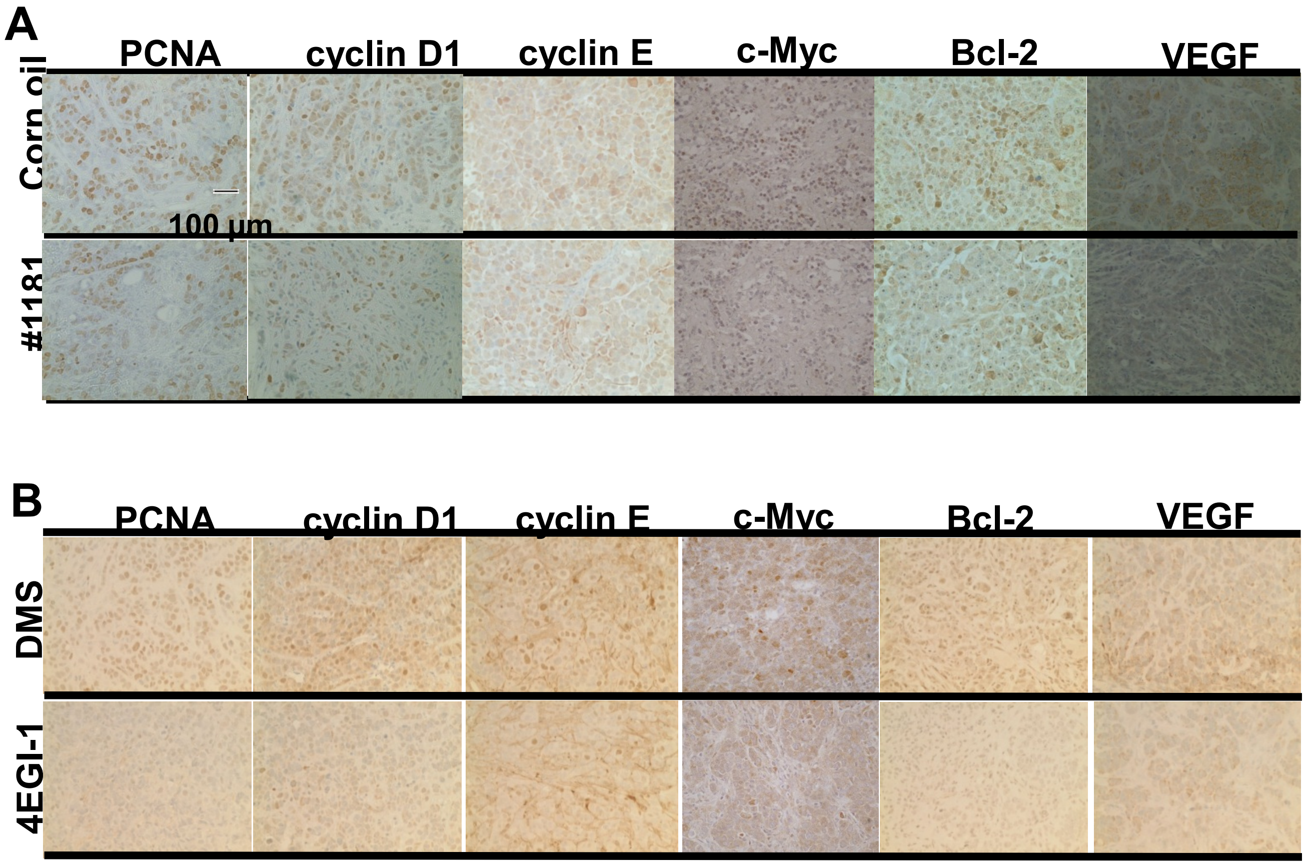

Figure S9: Compounds #1181 and 4EGI-1 down-regulate the expression of oncogenic proteins in xenograft models of human breast cancers. Human breast cancer xenografts MCF-7 (**A**) and CRL-1500 (**B**) were immunostained with antibodies specific for PCNA, cyclin D1, cyclin E, c-Myc, bcl-2, or VEGF. T he bar graph shown in Figure S13 is the quantification of immunostaining data quantified from three random fields from each sample section.

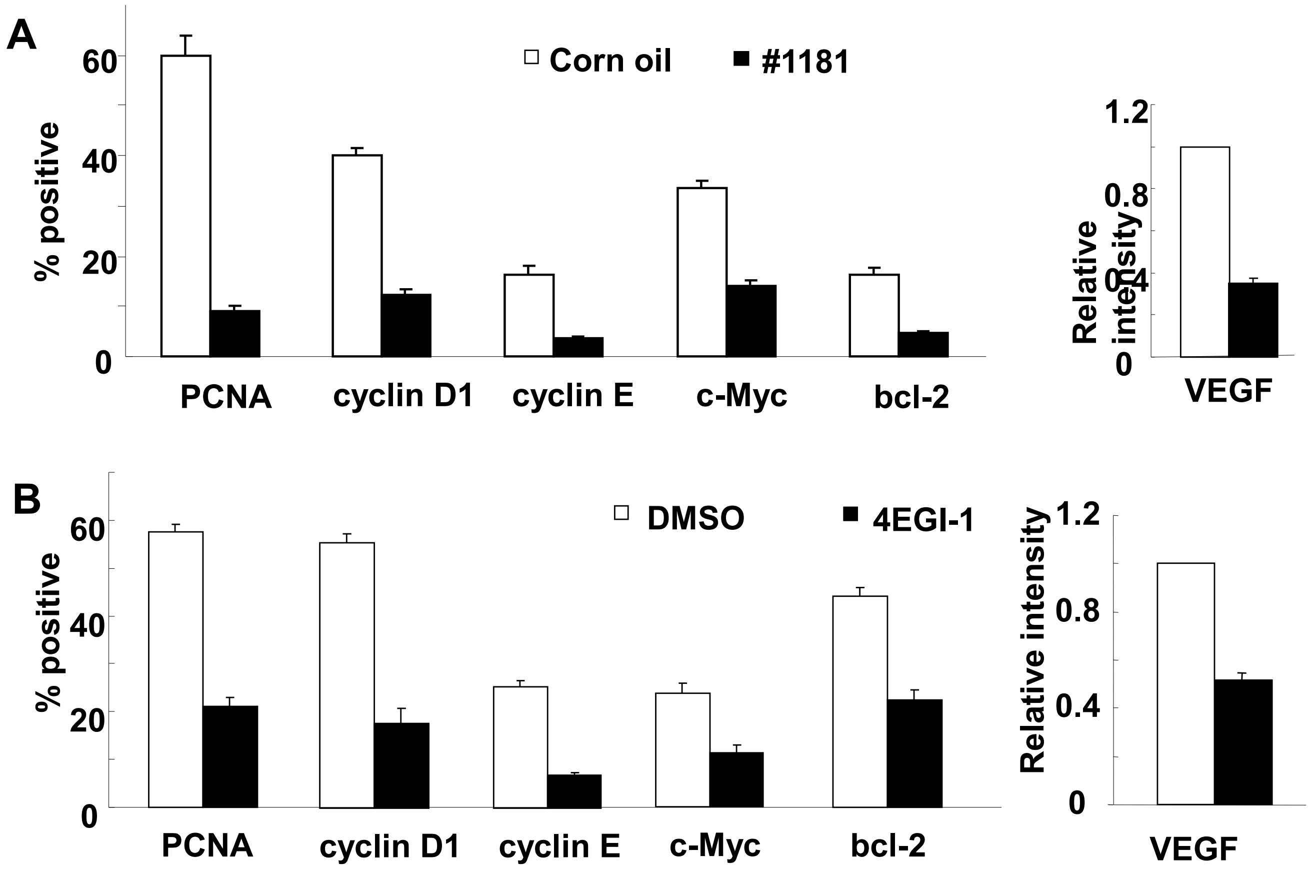

Figure S10: The quantification of immunohistochemistry for Figure S14. For quantifying the immunohistochemistry images, pictures were taken in three random fields from each sample section. The scoring was determined using ProImage software. The bars represent average signals of three independent fields and at least five different mice, error bars denote SEM.

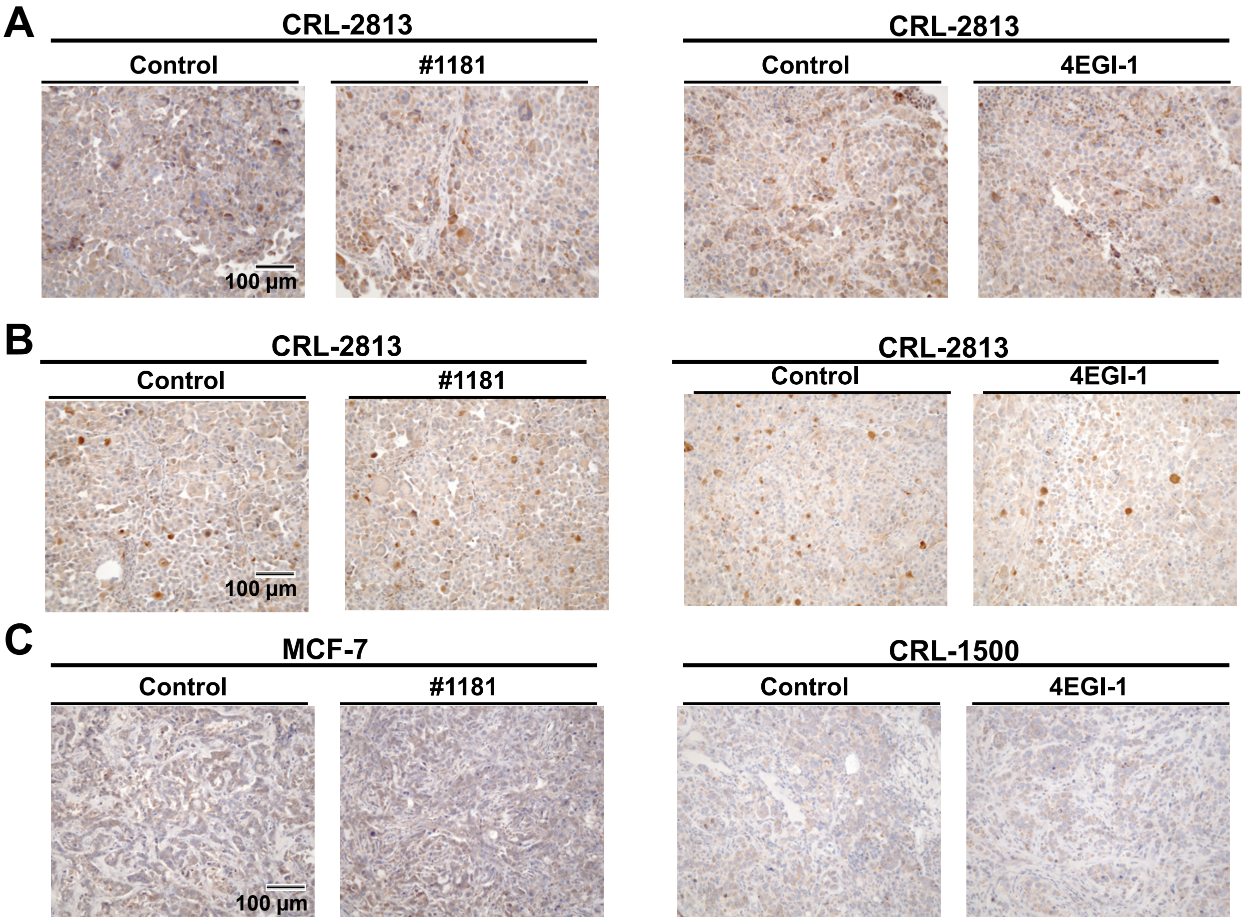

Figure S11. ***In vivo* evidence that the action of #1181 and 4EGI-1 is independent of B-raf, Erk1/2 and Akt. A** and **B)** CRL-2813 derived melanoma tumors from mice treated #1181 and 4EGI-1 or vehicle (from the efficacy studies) were sectioned and were immunostained with antibodies specific to B-raf **(A)**, phospho-Erk1/2 **(B)**. **C)** MCF-7 and CRL-1500 derived mammary tumors from mice treated with #1181 and 4EGI-1 or vehicle (from the efficacy studies) were sectioned and were immunostained with antibodies specific and phospho-Akt.
