# Supplementary material for: Tumor suppression by small molecule inhibitors of translation initiation
Source: Oncotarget. 2012 Aug 25;3(8):869–81. doi: 10.18632/oncotarget.598 (PMC3478463; doi:10.18632/oncotarget.598)
Supplement: Supplementary file 2 [file oncotarget-08-869-s002.docx]

TUMOR SUPPRESSION BY SMALL MOLECULE INHIBITORS OF TRANSLATION INITIATION - Limo Chen et al

**Supplementary Table S1**: Effects of #1181 and 4EGI-1 on Inhibition of Cancer Cell Proliferation

| **Cell Type** | **Tissue Origin** | **IC_50_ for # 1181(μM)** | **IC_50_ for 4EGI-1(μM)** |
| --- | --- | --- | --- |
|  |  |  |  |
| CCL121 | Fibrosarcoma | 12.2 | 4.5 |
| CCL257 | Lung cancer | 3.5 | >20 |
| CRL1435 | Prostate cancer | 10 | 18 |
| CRL1500 | Breast cancer | 13.5 | 3.2 |
| CRL1504 | Breast cancer | 3.4 | 5.3 |
| CRL1579 | Melanoma | 12 | 17.9 |
| CRL1676 | Melanoma | 12 | 4 |
| CRL1740 | Prostate cancer | 13.5 | 10.5 |
| CRL1848 | Lung cancer | 11 | 6 |
| CRL1902 | Breast cancer | 8 | 2.8 |
| CRL2314 | Breast cancer | 10.8 | 4.5 |
| CRL2321 | Breast cancer | 8 | 11.9 |
| CRL2351 | Breast cancer | 2.5 | 5.2 |
| CRL2500 | Melanoma | 11.5 | 17 |
| CRL2505 | Prostate cancer | 8 | 12.9 |
| CRL2808 | Melanoma | 3.7 | 6.5 |
| CRL2809 | Melanoma | 4 | 1.3 |
| CRL2813 | Melanoma | 2.3 | 4 |
| CRL2871 | Lung cancer | 9 | 8.2 |
| CRL5870 | Lung cancer | 1.8 | >20 |
| CRL5872 | Lung cancer | 10 | 3.8 |
| CRL5930 | Lung cancer | 9.8 | >20 |
| CRL7425 | Melanoma | 11.5 | 12 |
| CRL7568 | Melanoma | 6 | 1.3 |
| CRL7572 | Melanoma | 15 | >20 |
| HCC1395 | Breast cancer | 6.7 | 7.7 |
| HTB128 | Breast cancer | 10 | <0.54 |
| HTB174 | Lung cancer | 11.8 | 17.3 |
| HTB177 | Lung cancer | 11.9 | 6.6 |
| HTB179 | Lung cancer | 9 | 1.3 |
| HTB184 | Lung cancer | 9.8 | 17.4 |
| HTB22 | Breast cancer | 5.4 | 15.9 |
| HTB24 | Breast cancer | 10.7 | 5.7 |
| HTB26 | Breast cancer | 12.5 | >20 |
| HTB27 | Breast cancer | 8.3 | 8.9 |
| HTB30 | Breast cancer | 15 | 5.5 |
| HTB54 | Lung cancer | 10.8 | 18.5 |
| HTB55 | Lung cancer | 7.5 | 15 |
| HTB59 | Lung cancer | 11.7 | 18 |
| HTB70 | Melanoma | 12 | >20 |
| HTB72 | Melanoma | 15.5 | 17.5 |
| HTB81 | Prostate cancer | 12.5 | 13.1 |

**Supplementary Table S2:** Antibodies Used in the Study

| **Antibody** | **Catalog number** | **Sources** |
| --- | --- | --- |
| Akt (pan) | 4691L | Cell Signaling Technology |
| phospho-Akt (Ser 473) | 3787L | Cell Signaling Technology |
| α-tubulin | sc-5546 | Santa Cruz Biotechnology |
| β-actin | sc-1615 | Santa Cruz Biotechnology |
| Bcl-2 | 2876S | Cell Signaling Technology |
| b-FGF | 3196S | Cell Signaling Technology |
| B-Raf | ab33899 | Abcam |
| CHOP | sc-7351 | Santa Cruz Biotechnology |
| c-Myc | 9402S | Cell Signaling Technology |
| Cyclin D1 | 2978S | Cell Signaling Technology |
| Cyclin E | 4129S | Cell Signaling Technology |
| eIF4E | 2067S | Cell Signaling Technology |
| eIF4G | 2498S | Cell Signaling Technology |
| eIF2-α | sc-30882 | Santa Cruz Biotechnology |
| phospho-eIF2α | ab32157 | Abcam |
| Erk1/2 | 4695S | Cell Signaling Technology |
| phospho-Erk1/2 | 4376S | Cell Signaling Technology |
| 4E-BP1 | 9644S | Cell Signaling Technology |
| p4E-BP1 (Thr 37/46) | 2855L | Cell Signaling Technology |
| p4E-BP1 (Ser 65) | 9451S | Cell Signaling Technology |
| p4E-BP1 (Thr 70) | 9455S | Cell Signaling Technology |
| mTOR | 2983S | Cell Signaling Technology |
| PCNA | sc-7907 | Santa Cruz Biotechnology |
| Survivin | 2808S | Cell Signaling Technology |
| Ubiquitin | sc-8017 | Santa Cruz Biotechnology |
| VEGF | AF564 | R & D Systems |
